# Ethanol Extract of Sanguisorbae Radix Inhibits Mast Cell Degranulation and Suppresses 2,4-Dinitrochlorobenzene-Induced Atopic Dermatitis-Like Skin Lesions

**DOI:** 10.1155/2016/2947390

**Published:** 2016-03-15

**Authors:** Ju-Hye Yang, Jae-Myung Yoo, Won-Kyung Cho, Jin Yeul Ma

**Affiliations:** Korean Medicine (KM) Application Center, Korea Institute of Oriental Medicine, 70 Cheomdan-ro, Dong-gu, Daegu 41062, Republic of Korea

## Abstract

Sanguisorbae Radix (SR) is well known as herbal medicine named “Zi-Yu” in Korea, which is the dried roots of* Sanguisorba officinalis* L. (Rosacease). We investigated the underlying mechanism on the inhibition of atopic dermatitis (AD) of an ethanol extract of SR (ESR) using 2,4-dinitrochlorobenzene- (DNCB-) induced AD mice model. Oral administration of ESR significantly suppressed DNCB-induced AD-like symptoms such as scratching behavior, ear thickness, epidermal thickness, and IgE levels. To investigate the effects of ESR treatment on degranulation of IgE/Ag-activated mouse bone marrow-derived mast cells (BMMCs), we measured the release of *β*-hexosaminidase (*β*-HEX, degranulation marker). ESR decreased the infiltration of eosinophils and mast cells into the AD skin lesions. Furthermore, ESR significantly inhibited degranulation of IgE/Ag-activated BMMCs. We have demonstrated that ESR decreased AD symptoms in mice and inhibits degranulation of IgE/Ag-activated mast cells. Our study suggests that ESR may serve as a potential therapeutic candidate for the treatment of AD symptoms.

## 1. Introduction

Atopic dermatitis (AD) is a chronic inflammatory skin disease. AD causes epidermal thickness with cutaneous hypersensitivity associated with increased serum immunoglobulin E (IgE) levels and infiltration of inflammatory cell types including mast cells and eosinophils [1, 2]. AD is a complex interaction of innate and adaptive immune responses based on an individual's genetic, environmental, pharmacological, and psychological conditions.

IgE secretion is an important characteristic of AD, with elevated levels related to disease severity in AD patients. Previous studies report that high serum IgE levels induce activation of mast cells and cause an allergic reaction [2, 3]. Because mast cells aggregate high-affinity IgE receptors (Fc*ε*RI) on their surfaces, which is important in the proinflammatory/allergic response, mast cells are believed to play an important role in the induction of AD [4, 5]. An association between mast cell activation and AD is suggested by the increase in mast cell counts and activation in AD lesions [6]. In addition, mast cells produce inflammatory mediators such as prostaglandin D_2_ (PGD_2_) and induce eosinophil chemotaxis to inflammatory sites in the skin of AD patients [7].

Sanguisorbae Radix (SR) is well known as herbal medicine named “Zi-Yu” in Korea, which is the dried roots of* Sanguisorba officinalis* L. (Rosacease) [8]. SR has been used as a traditional herbal medicine to treat diarrhea, chronic intestinal infections, duodenal ulcers, internal hemorrhage, and burns [8–10]. SR includes saponin glycosides and ellagitannins (i.e., pomolic acid, sanguisorbic acid dilactone, and ziyuglycoside I). SR and its active components have been demonstrated to have biological activity* in vivo* and* in vitro*. SR inhibits the renal dysfunction induced by lipopolysaccharide (LPS) endotoxin* in vivo* by suppressing the serum nitrite/nitrate levels and the activity of inducible nitric oxide synthase (iNOS) [11]. In addition, SR was reported to have antioxidative stress and antiaging activity* via* suppressing of nitric oxide (NO) production and activity of iNOS [10, 12, 13]. SR blocked H_2_O_2_-induced ROS generation* in vitro* and MCAO-induced ischemic brain damage* in vivo* [14]. Additional reported properties of SR include anticancer [15], antiasthma [16], anticoronavirus [17], and antiwrinkle activity [18]. Many researchers reported anti-inflammatory activities of SR. Nonetheless, antiatopic dermatitis effects of SR are not reported. Recently, studies reported inhibitory effect of SR on contact dermatitis [19], and we reported anti-inflammatory mechanism of ESR in human keratinocyte by suppressing the expression of TNF-*α*/IFN-*γ*-stimulated chemokines and proinflammatory molecules via blockade of NF-*κ*B, STAT-1, and ERK activation. However, the biological and pharmacological actions of SR are not fully understood in atopic dermatitis.

In this study, we examined the effects of ESR on 2,4-dinitrochlorobenzene- (DNCB-) induced AD mouse skin lesions and mouse bone marrow-derived mast cells (BMMCs) to determine its therapeutic potential for the treatment of AD.

## 2. Materials and Methods

### 2.1. Preparation of ESR

SOL roots were obtained from Yeongcheon Oriental Herbal Market (Yeongcheon, Korea). All samples were deposited in the herbal bank of KM Application Center, Korea Institute of Oriental Medicine (KIOM; Daejeon, Republic of Korea). To prepare the ESR, dried SR pieces (50.0 g) were extracted using 390 mL 70% ethanol in a 40°C shaking incubator for 24 h. We obtained 9.5 g, giving us a yield of 19.1%.

### 2.2. Animals

Male BALB/c mice (5 weeks old) were purchased from Samtako Bio Korea (Osan, Korea). Mice were observed every day for one week during quarantine and acclimation. Mice were divided into six groups (*n* = 5 per group): (1) negative control (vehicle), (2) DNCB + vehicle (control), (3) DNCB + 50 mg/kg ESR, (4) DNCB + 100 mg/kg ESR, (5) DNCB + 200 mg/kg ESR, and (6) and 1 mg/kg dexamethasone (Dexa.). All groups were maintained under standard conditions of temperature (22.5 ± 0.5°C), humidity (42.6 ± 1.7%), 12 h lighting (8:00 AM–8:00 PM, 290 lx), ventilation (10–15 times per hour), and diet (Teklad Global Diets, Harlan Laboratories Inc., USA). This study was conducted according to the guidelines listed in the Pharmaceutical Affairs Act of Korea Food and Drug Association (KFDA) and approved by the Institutional Animal Care and Use Committee of Korea Conformity Laboratories (IA11-00920).

### 2.3. Induction of AD and Drug Treatment

**Figure 1:**
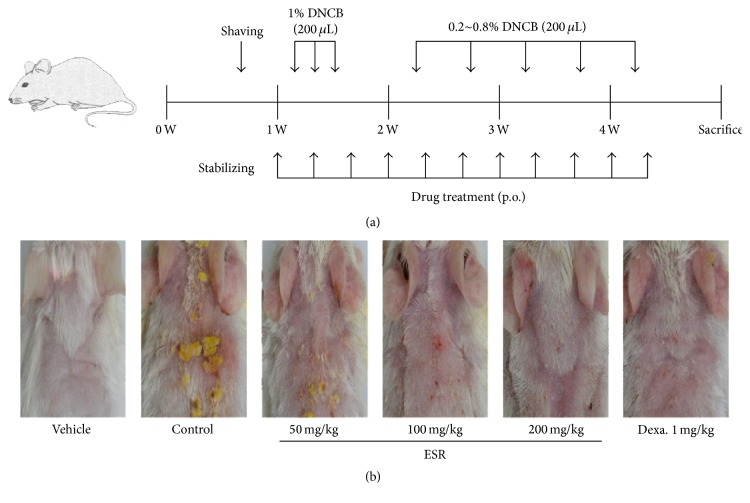
Effects of ESR on the development of DNCB-induced AD mouse skin lesions in BALB/c mice. (a) Experimental schedule for the induction of dermatitis. (b) Effects of ESR on clinical features of DNCB-induced AD skin lesions. Vehicle, negative control; control, DNCB + vehicle; ESR, DNCB + ESR treated group (50, 100, and 200 mg/kg); Dexa., DNCB + 1 mg/kg dexamethasone treated group.

After 1 week of acclimation, DNCB was applied to the dorsal skin and both ears of BALB/c mice to induce AD-like symptoms and skin lesions. One day after complete dorsal hair removal, 200 *μ*L 1% DNCB dissolved in an acetone : olive oil mixture (3 : 1 vol/vol) was applied to the dorsal skin and 20 *μ*L was applied to both ears (days 2–4). Five days after dorsal hair removal, 0.2–0.8% DNCB dissolved in an acetone : olive oil mixture (3 : 1 vol/vol) was applied to challenge the dorsal skin (200 *μ*L) and both ears (20 *μ*L each) two times a week for 2 weeks. Similarly, 1% DNCB solution was applied one day prior to sacrifice. ESR dissolved in saline (10 mL/kg body weight) was orally administered by gavage at 50, 100, and 200 mg/kg or using dexamethasone (Sigma-Aldrich, St. Louis, MO, USA; 1 mg/kg) three times a week for 4 weeks (days 0–24). The experimental scheme is summarized in Figure 1(a).

### 2.4. Scratching Behavior and Ear Thickness Measurements

Scratching behavior was measured by placing each mouse into a cage once a week for 10 min and observing and recording behavior [20]. For each mouse, ear thickness was measured and recorded with a micrometer (Mitutoyo, Kawasaki, Japan). To minimize variation, a single investigator performed all measurements [21].

### 2.5. Histopathological Analysis

At the end of the study period, the dorsal skin lesions of each mouse were removed, fixed with 10% neutral-buffered formalin, and embedded in paraffin. 4 *μ*m thick sections were stained with hematoxylin and eosin (H&E) and toluidine blue to detect epidermal thickness and inflammatory cells (i.e., eosinophils and mast cells), respectively. Histopathological evaluation of all skin sections occurred in a blind fashion [1]. All samples were observed using an inverted microscope and data are representative of five observations (Nikon Eclipse Ti, Nikon, Tokyo, Japan).

### 2.6. Serum IgE Measurements

At the end of the study period, mice were sacrificed and whole blood was collected. Blood samples were centrifuged at 2,000 ×g for 20 min at 4°C. Serum obtained from whole blood was stored at −80°C until use. Serum IgE levels were measured using enzyme-linked immunosorbent assay (ELISA) kits according to the manufacturer's instructions (BioLegend, San Diego, CA, USA). Inhibitory effects of ESR were determined based on absorbance at 450 nm measured using a multilabel microplate reader (SpectraMax i3, Molecular Devices, Silicon Valley, CA, USA).

### 2.7. BMMCs

BMMCs isolated from male BALB/c mice were cultured for up to 10 weeks in RPMI-1640 media containing 2 mM L-glutamine, 0.1 mM nonessential amino acids, antibiotics, and 10% fetal bovine serum (FBS) with 20% pokeweed mitogen-stimulated spleen condition medium (PWM-SCM) as a source of interleukin-3 (IL-3). After 3 weeks, more than 98% of the cells were verified as BMMCs according to a previously described procedure [22].

### 2.8. Cell Viability Assay

Cell cytotoxicity was analyzed using a cell counting kit (CCK) (Dojindo Molecular Technologies, Inc., Kumamoto, Japan). BMMCs (2 × 10^5^ cells/well) were seeded into 96-well plates. After 24 h, ESR was added at concentrations of 10, 50, 100, and 200 *μ*g/mL, and plates were incubated for 24 h at 37°C in a 5% CO_2_ incubator. CCK solutions were added to each well and cells were incubated for 1 h. Optical density was measured at 570 nm using an ELISA plate reader (Infinite M200, Tecan, Männedorf, Switzerland).

### 2.9. *β*-HEX Release Assay

*β*-hexosaminidase (*β*-HEX), a marker of mast cell degranulation, was quantified by spectrophotometric analysis of the hydrolysis of p-nitrophenyl-2-acetamido-2-deoxy-*β*-D-glucopyranoside (4-Nitrophenyl N-acetyl-*β*-D-glucosaminide (p-NAG), Sigma-Aldrich, St. Louis, MO, USA).

For cell stimulation, BMMCs (5 × 10^5^ cells/mL) were sensitized overnight with 100 ng/mL anti-dinitrophenyl (DNP) antibody and then stimulated for 15 min with 25 ng/mL DNP-human serum albumin (HSA). To investigate the effects of ESR, ESR of varying concentrations was added 1 h prior to the addition of DNP-HSA. After harvesting supernatants according to a previously described procedure [23], the percentage of *β*-HEX released into the supernatant was calculated using the following formula: [supernatant/(supernatant + pellet)] × 100.

### 2.10. Statistical Analysis

Data were analyzed using GraphPad Prism software (ver. 5.0 GraphPad Software, San Diego, CA, USA). Results are expressed as the mean ± standard error of the mean (SEM) and were evaluated using Student's* t*-test or analysis of variance (ANOVA). A *p* value of less than 0.05 was considered statistically significant.

## 3. Results

### 3.1. Effects of ESR on the Development of DNCB-Induced AD Mouse Skin Lesions

To investigate the therapeutic effects of ESR on AD mouse lesions, we administered ESR following the induction of AD mouse skin lesions using DNCB. Topical application of DNCB-induced crusting, epidermal thickness, redness, and dryness of skin are shown in Figure 1(b). However, skin conditions significantly improved in ESR-administered groups compared to the control group.

### 3.2. Effects of ESR on Scratching Behavior and Ear Thickness in AD Mice

**Figure 2:**
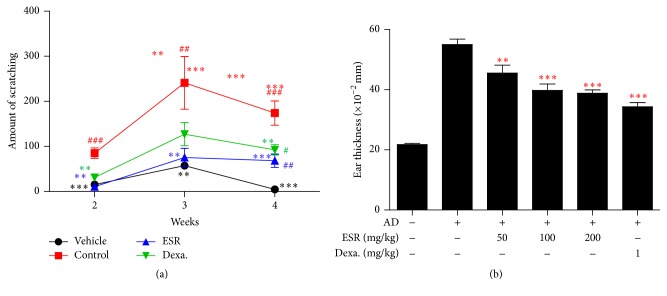
Effects of ESR on scratching behavior and ear thickness in AD mouse skin lesions. (a) Experimental induction of AD mouse skin lesions. (b) Ear thickness was measured with a dial thickness gauge. Results are expressed as the mean ± standard error of the mean (SEM) (*n* = 5). Vehicle, negative control; control, DNCB + vehicle; ESR, DNCB + 200 mg/kg ESR; Dexa., DNCB + 1 mg/kg dexamethasone treated group. ^#^ *p* < 0.05, ^##^ *p* < 0.01, and ^###^ *p* < 0.001 versus vehicle; ^*∗∗*^ *p* < 0.01 and ^*∗∗∗*^ *p* < 0.001 versus control.

Scratching behavior of the control group was rapidly increased and became significantly different from that observed in the vehicle group at day 14 after DNCB application. In AD mice treated with 200 mg/kg ESR, scratching was inhibited strongly compared to the vehicle group (Figure 2(a)). In addition, ESR significantly reduced DNCB-induced ear thickness in a dose-dependent manner. These results suggest that ESR has a therapeutic effect that can reduce AD symptoms in mice.

### 3.3. Effects of ESR on Dorsal Skin Thickness and Eosinophil Accumulation in AD Mouse Skin Lesions

**Figure 3:**
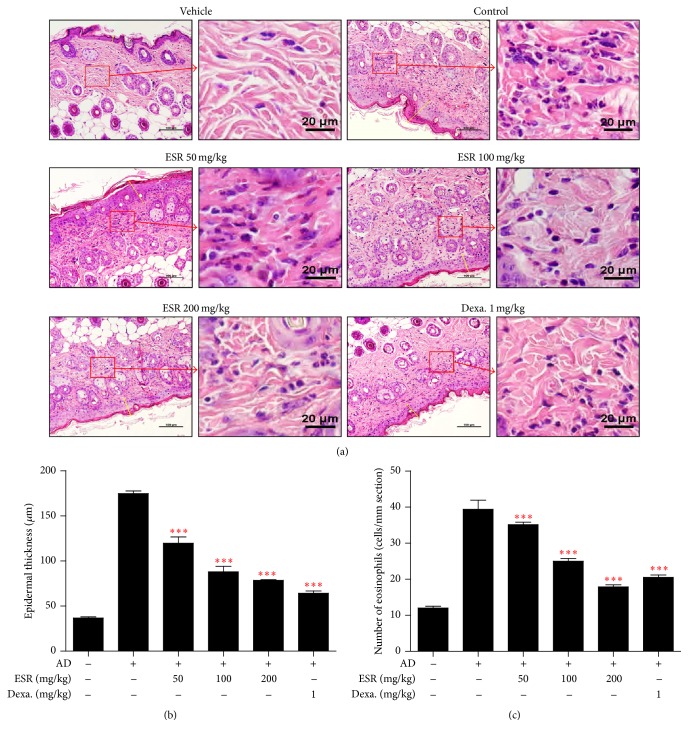
Effects of ESR on epidermal thickness and eosinophil accumulation in DNCB-induced AD mouse skin lesions. (a) H&E stained AD mouse skin lesions (20x, scale bar = 100 *μ*m) and image of amplification sections (right image of group, scale bar = 20 *μ*m). (b) Determination of epidermal thickness. (c) Number of eosinophils per mm section. Epidermal thickness in H&E stained sections was measured under a microscope and the number of eosinophils accumulations is expressed as the average total count in five fields of 100 *μ*m^2^. ^*∗∗∗*^ *p* < 0.001 versus control.

Multiple applications of DNCB-induced infiltration of high levels of inflammatory cells into the skin result in increased dermal thickness [24]. To determine whether ESR treatment decreases eosinophil accumulation in AD mouse skin lesions, we performed H&E staining on the skin following oral administration of ESR and observed the tissue under an optical microscope. Dorsal histopathological results showed thickening of the epidermis and eosinophil accumulation within the dorsal skin lesions of BALB/c mice with AD. The treatment of 100 and 200 mg/kg ESR suppressed eosinophil accumulation and reduced dorsal skin thickness in a dose-dependent manner (Figures 3(a) and 3(b)).

### 3.4. Effects of ESR on Mast Cell Infiltration and Serum IgE Levels in AD Mice

**Figure 4:**
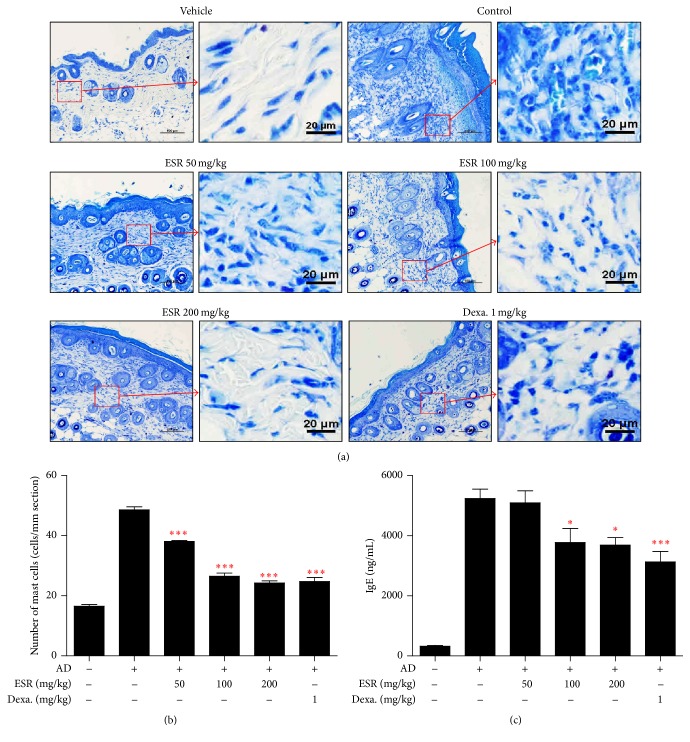
Effects of ESR on mast cell infiltration and serum IgE level in DNCB-induced AD mouse lesions. (a) Toluidine blue stained AD mouse skin lesions (20x, scale bar = 100 *μ*m) and image of amplification sections (right image of group, scale bar = 20 *μ*m). (b) Number of mast cells per mm section. Mast cell infiltration in toluidine blue stained sections is expressed as the average total count in five fields of 100 *μ*m^2^. ^*∗*^ *p* < 0.05, ^*∗∗∗*^ *p* < 0.001 versus control.

Elevated serum IgE levels and mast cell infiltration are major characteristics of AD and related to disease severity [24]. To assess the effects of ESR on mast cell infiltration, we stained sliced cross sections of skin lesions with toluidine blue. As shown in Figure 4, we found an increase in mast cell infiltration (Figures 4(a) and 4(b)) and IgE levels in DNCB-treated mice compared to the vehicle group (Figure 4(c)). However, ESR significantly decreased the level of IgE elevated by DNCB induction and inhibited mast cell infiltration, in a dose-dependent manner (Figures 4(b) and 4(c)).

### 3.5. Effects of ESR on BMMC Cytotoxicity and Mast Cell Degranulation

**Figure 5:**
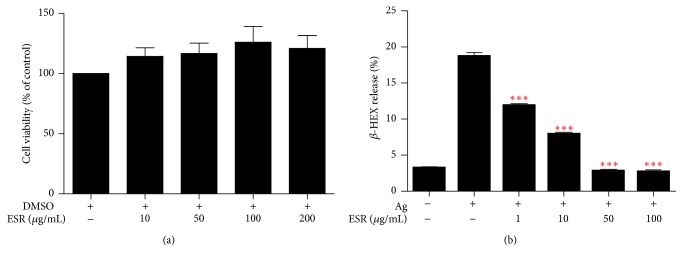
Effects of ESR on cytotoxic and mast cell degranulation in BMMCs. (a) Cytotoxicity of BMMCs was determined using a CCK assay. BMMCs were seeded into 96-well plates and treated with various concentrations of ESR for 24 h. (b) Inhibitory effects of ESR on *β*-HEX release in BMMCs. BMMCs were sensitized overnight with anti-DNP IgE and challenged with DNP-HAS (Ag) with or without ESR. The data are presented as the mean ± SEM of three independent experiments. ^*∗∗∗*^ *p* < 0.001 versus control.

Degranulation of mast cells is correlated with AD severity and the recruitment of immune cells to inflammatory sites in AD patients [2]. To investigate the effect of ESR treatment on degranulation of IgE/Ag-activated BMMCs, we measured the release of *β*-HEX in the presence or absence of ESR. As shown in Figure 5(b), ESR potently reduced *β*-HEX release in a dose-dependent manner.

To check cytotoxicity of ESR on BMMCs, the cells were incubated with different ESR concentrations (10, 50, 100, and 200 *μ*g/mL) for 18 h and subjected for CCK-8 assay. ESR at 200 *μ*g/mL had no significant cytotoxic effect on BMMCs after 24 h (Figure 5(a)).

## 4. Discussion

AD is an inflammatory disease characterized by an increase of cells associated with a Th2 response, including monocytes, macrophages, eosinophils, and mast cells. The pathogenesis of AD is primarily driven by Th2 immune responses and increased IgE production [5]. Proliferation, migration, and local activation of eosinophils are common in AD. Eosinophils act as immunoregulatory factors by secreting a variety of cytokines and chemokines attracting more eosinophils to the site of inflammation. Furthermore, eosinophils promote a switch from acute to chronic responses in AD [2]. ESR treatment reduces eosinophil accumulation in AD mouse skin lesions (Figures 3(a) and 3(c)).

High levels of serum IgE represent another characteristic of AD; thus, it is likely that targeting IgE may impact AD disease outcome. Specifically, IgE binding to mast cells affects the development and severity of AD [21]. Mast cells play a key role in allergic reactions via the production and secretion of proinflammatory mediators such as histamine, chemokines, cytokines, and growth factors. On the surface of mast cells, Th2 cells produce IgE, which binds to the Fc*ε*RI on the mast cell surface [25]. Fc*ε*RI-mediated mast cell activation is triggered by antigen IgE cross-linking and leads to the degranulation and expression of proinflammatory mediators. Fc*ε*RI-activated mast cells induce IgE elevation and increase mast cells in a majority of AD patients; therefore, mast cells are hypothesized to contribute to the pathogenesis of AD [3]. Our findings show that ESR treatment suppresses serum IgE levels and mast cell infiltration in a DNCB-induced AD mouse model.

*β*-HEX, a degranulation marker, is released along with other proinflammatory mediators when mast cells are activated [23, 26]. Therefore, we examined the degranulation by measuring *β*-HEX release in antigen IgE-activated BMMCs and confirmed that ESR treatment inhibited *β*-HEX release in a dose-dependent manner.

Active components of SR include phenolic compounds including tannins and flavonoids, saponin glycosides, and ellagitannins (i.e., pomolic acid, sanguisorbic acid dilactone, and ziyuglycoside I) [27]. Ziyuglycoside I and ziyuglycoside II are the major effective ingredients of triterpenoid saponins extracted from* Sanguisorba officinalis* L., and many studies have focused on their pharmacological activities. In addition, ziyuglycoside I is a major marker of SR to confirm the origin of the medicinal plant in Korean Pharmacopoeia. Thus, we used ziyuglycoside I as a phytochemical marker of SR in the experiment (data not shown).

In this study, we investigated the therapeutic effects of ESR in the treatment of AD using a DNCB-induced AD mouse model and BMMCs. ESR significantly improved scratching behavior, ear thickness, epidermal thickness, and serum IgE levels in DNCB-induced AD mice. We observed a reduction in the infiltration of eosinophils and mast cells into the AD skin lesions following ESR treatment. In addition, ESR significantly inhibited degranulation of IgE/Ag-activated BMMCs. The therapeutic effects of ESR treatment on AD are supported by the beneficial effect of anti-IgE therapy in a number of clinical studies [2, 3].

In conclusion, these results demonstrate that ESR decreases AD symptoms in mice and inhibits degranulation of IgE/Ag-activated mast cells. Our study suggests that ESR may serve as a potential therapeutic candidate for the treatment of AD.

## Abbreviations

ESR: Ethanol extract of Sanguisorbae Radix
AD: Atopic dermatitis
DNCB: 2,4-Dinitrochlorobenzene
H&E: Hematoxylin and eosin
Fc*ε*RI: High-affinity immunoglobulin E receptor
BMMCs: Mouse bone marrow-derived mast cells
*β*-HEX: *β*-Hexosaminidase.
